# Protective Effects of Combined Utilization of Quercetin and Florfenicol on Acute Hepatopancreatic Necrosis Syndrome Infected *Litopenaeus vannamei*

**DOI:** 10.3390/antibiotics11121784

**Published:** 2022-12-09

**Authors:** Qianqian Zhai, Zhiqiang Chang, Jitao Li, Jian Li

**Affiliations:** 1 Key Laboratory for Sustainable Utilization of Marine Fisheries Resources, Ministry of Agriculture and Rural Affairs, Yellow Sea Fisheries Research Institute, Chinese Academy of Fishery Sciences, Qingdao 266071, China; 2 Function Laboratory for Marine Fisheries Science and Food Production Processes, Qingdao National Laboratory for Marine Science and Technology, Qingdao 266237, China

**Keywords:** immunomodulation, VP_AHPND_, *Litopenaeus vannamei*, quercetin, florfenicol

## Abstract

This study aimed to determine the immunity, survival rate, and disease resistance of *Litopenaeus vannamei* treated using quercetin and florfenicol alone or in combination, after infection with acute hepatopancreatic necrosis syndrome caused by *Vibrio parahaemolyticus* (VP_AHPND_). After infection with VP_AHPND_, different types of feed were given to the shrimp for 5 days, including a control diet (drug-free), florfenicol only diet (15 mg/kg), quercetin only diet (400 mg/kg), a low-dose florfenicol/quercetin combined diet (200 mg/kg quercetin + 7.0 mg/kg florfenicol), a moderate-dose florfenicol/quercetin combined diet (400 mg/kg quercetin + 15 mg/kg florfenicol), and a high-dose florfenicol/quercetin combined diet (800 mg/kg quercetin + 30 mg/kg florfenicol). The cumulative mortality of shrimp was significantly reduced in the drug combination groups compared with either drug used alone (*p* < 0.05). The density of Vibrio was significantly lower and the immune parameters were significantly increased in the drug combination groups compared with either drug used alone (*p* < 0.05). Moreover, in the drug combination groups, the hepatopancreas tubules showed better integrity and structure compared with those when either drug was used alone. Therefore, compared with single drug treatment, the florfenicol and quercetin combination enhanced disease resistance, survival, and immune activity of VP_AHPND_-infected shrimp. When the combination treatment is used, the dosage of florfenicol can be reduced and a better therapeutic effect is obtained.

## 1. Introduction

As the main aquacultured shrimp worldwide, *Litopenaeus vannamei* (*Penaeus vannamei*) is also the most produced shrimp in China [1,2]. However, *L. vannamei* is facing the threat of various large-scale bacterial and viral disease outbreaks because of the accelerated development of intensive farming, and the environmental destruction caused by it. For example, in recent years, shrimp production has been severely threatened by acute hepatopancreatic necrosis disease (AHPND), a new bacterial disease caused mainly by *Vibrio parahaemolyticus* (VP_AHPND_). VP_AHPND_ cells contain Pir toxin-like proteins encoded by two genes (pirA- and pirB-like), whose activities cause severe mortality in farmed shrimp [3]. Studies have reported that AHPND causes damage to shrimp hepatopancreas cells via the proliferation of VP_AHPND_ and Pir secretion into the shrimp’s stomach [4].

Up to now, the prevention and control of shrimp disease has employed a variety of methods, among which the most widely used and effective method is the use of antibiotics [5]. Antibiotic overuse can cause drug residues and environmental pollution, leading to the emergence of bacterial resistance [6,7]. There is increasing public concern about the overuse of antibiotics, prompting people to look for safer and more environmentally friendly drugs. Under these circumstances, traditional Chinese medicine has attracted attention because of its environmental friendliness and safety [8,9]. Moreover, the resources for Chinese traditional medicines are abundant and easy to obtain, and their content of active substances is rich [10,11]. Chinese herbal medicines and antibiotics work in different ways, such that their combinations can improve the treatment effect of various diseases and can decrease the emergence of antibiotic resistance [12]. Therefore, to produce an improved therapeutic effect and ameliorate the damage resulting from the use of large amounts of antibiotics, combinations of antibiotics and Chinese herbal medicines have become a research focus in recent years.

As a third generation of chloramphenicol antibiotic, florfenicol mainly exerts antibacterial effects by binding the bacterial 50 s ribosomal subunit [13]. Florfenicol is non-toxic, shows great antibacterial activity, and has few side effects; therefore, it is used widely to prevent and treat aquaculture animal diseases [14]. As a major class of phytochemicals (flavonoids), quercetin is found in high levels in fruits and vegetables, such as cherries, red grapes, apples, onions, broccoli, cabbage, kale, and tea [15,16,17]. Quercetin has many biological activities such as anti-inflammatory, antioxidant, anti-tumor, and immune activities [18,19]. Adding quercetin to the diets of aquaculture animals improved their immunity and antioxidant capacity [20,21,22]. Thus, aquaculture animals’ disease resistance could involve both florfenicol and quercetin. However, whether these two drugs in combination could increase shrimp disease resistance compared with either drug used alone, or allow the dose reduction of florfenicol, is not yet known.

Therefore, this study aimed to investigate the effect of the combination of florfenicol and quercetin on *L. vannamei* immunity, survival, and the morphology of the hepatopancreas after VP_AHPND_ infection. We hope that our findings will provide a practical basis to treat bacterial diseases of aquaculture animals using combinations of antibiotics and Chinese herbal medicine.

## 2. Materials and Methods

### 2.1. Experimental Diets and Treatment

Qingdao Debon Pharmaceutical Company (Qingdao, China) provided florfenicol (purity ≥ 98%). Shanghai Yuanye Biological Technology Co., Ltd. (Shanghai, China) provided quercetin (purity > 98%).

Table 1 details the formulation of the control feed and various medicated (florfenicol and quercetin) feeds used in the experiment. To ensure the growth of *L. vannamei*, the basic diet contained 431 g/kg crude protein and 73 g/kg crude fat. Florfenicol and quercetin at various doses replaced cellulose in the feed in the following experimental groups: VP_AHPND_ infection only group (0 mg/kg), quercetin only group (400 mg/kg), florfenicol only group (15 mg/kg), low-dose combination (LDC) group (200 mg/kg quercetin and 7.0 mg/kg florfenicol), moderate-dose combination (MDC) group (400 mg/kg quercetin and 15 mg/kg florfenicol), high-dose combination (HDC) group (800 mg/kg quercetin and 30 mg/kg florfenicol), and control group (0 mg/kg) [23,24,25]. The ingredients were ground and filtered through a 200 μm filter. Thereafter, the mixed powder was combined with tap water and fish oil to make a dough. Finally, a meat grinder was used to extrude the dough, which was heated for 30 min at 90 °C, dried without light, formed into pellets, and kept at −20 °C for subsequent experiments.

### 2.2. Bacterial Culture

*L. vannamei* displaying symptoms of AHPND, the source of the VP_AHPND_ strain, (No. 20130629002S01) were kindly provided by Yellow Sea Fisheries Research Institute (Qingdao, China) and cultured as described previously [25]. The bacterial plate counting method was used to assess the bacterial abundance in the culture media. A final VP_AHPND_ density of 10^7^ CFU/mL was obtained by serial dilutions in PBS.

### 2.3. Experimental Animals

Qingdao Ruiz Group Co., Ltd., Qingdao, China, provided the experimental shrimp used in this study, which weighed 4.55 ± 0.26 g. Before the experiment, the shrimp were acclimated for one week in 200 L plastic reservoirs and fed with the basic diet, under controlled breeding environment conditions of pH 8.1 ± 0.1, water temperature 24 ± 0.5 °C, and salinity 33%.

### 2.4. Experiment Design and Sampling

The 1365 shrimp were divided into 3 batches. Batch 1 (350 shrimp) was divided into 7 experimental groups as defined in Section 2.1, with 50 shrimp in each group. The experiment was set up in three replicates. Except for the control group, the other 6 groups received 10 μL of VP_AHPND_ intramuscularly at the concentration of 10^7^ CFU/mL (the 24 h half lethal concentration). Then, all the groups received the appropriate feed (Table 2) immediately. At 0, 0.5, 1, 3, and 5 days post infection (dpi), we recorded the RPS of the drugs and the cumulative shrimp mortality rate. Batch 2 (105 shrimp) were divided into the same 7 groups (*n* = 15 per group). On the fifth dpi, the 7 groups of shrimp were anatomized to obtain their hepatopancreases. The hepatopancreas samples were subjected to fixation using Davidson solution before histological analysis. Batch 3 (210 shrimp) were split into the same 7 groups and used to assess the VP_AHPND_ hepatopancreas density and to evaluate the immune parameters. From each group of shrimp, 6 were selected randomly at 0, 0.5, 1, 3, and 5 dpi, and pairs of shrimp were used as one specimen; therefore, at each sampling time there were 3 specimens in each group. A syringe containing anticoagulant was used to extract the shrimp hemolymph. The hemolymph was separated into 2 parts: one was used to calculate the total hemocyte count and the hemocyanin’s concentration; the other was centrifuged at 3000× *g* for 10 min to separate the hemocyte from the plasma. The obtained plasma was stored at −20 °C for later determination of the immune enzyme and antibacterial activities. The RNA was prepared from the obtained hemocyte using Trizol reagent for detecting immune-related gene expression.

### 2.5. Shrimp Survival Assay

The dead shrimp in each group were recorded at 0, 0.5, 1, 3, and 5 dpi. to calculate the cumulative mortality (%) and the (RPS, %) were calculated as described previously [25].

### 2.6. The Density of VP_AHPND_ in Hepatopancreas

The obtained hepatopancreas from each group were ground in PBS in a sterile environment. Then, 100 μL of the ground hepatopancreas tissue solution was spread on a thiosulfate–citrate–bile-salt–sucrose (TCBS) agar plate, cultured at 28 °C for 24 h, and the colonies were counted to calculate the hepatopancreas VP_AHPND_ density.

### 2.7. Immune Parameters Assessment

#### 2.7.1. Total Hemocyte Counts

Under a light microscope, a hemocytometer was used to count the hemolymph cells mixed with the anticoagulant to calculate the total hemocyte count (THC, cells/mL hemolymph). The anticoagulant was formulated as Zhai et al. described [25].

#### 2.7.2. Hemocyanin Determination

To determine the concentration of hemocyanin (HEM), 100 μL of the mixture of hemolymph and anticoagulant was mixed with 900 μL sterile water and measured according to the method used previously [25].

#### 2.7.3. Antibacterial Activity

Bacterial colonies on solid medium were washed off using sterile PBS and collected. The technique of Hultmark et al. was used to determine the antibacterial activity of the hemolymph [26].

#### 2.7.4. Activities of Immunity-Related Enzymes

The shrimp acellular hemolymph phenol oxidase (PO) activity was determined according to a previously reported method [27]. Superoxide dismutase (SOD), glutathione peroxidase (GSH-Px), iysozyme (LZM), alkaline phosphatase (AKP) and acid phosphatase (ACP) activities were measured using commercial kits (Institute of Biological Engineering, Nanjing, China). The Bradford method was used to determine the protein content in hemolymph.

#### 2.7.5. Expression of Immunity-Related Genes

Total hemocyte RNA was isolated and a real-time PCR was performed to detect expression of immunity-associated genes including *Tlr* (encoding toll-like receptor), *Lzm*, *Lec* (encoding lectin), *Cru* (encoding crustin), *CatB* (encoding cathepsin B), and *Alf* (encoding anti-lipopolysaccharide factor) as described previously [28]. The primers for PCR are listed in Table 2.

### 2.8. HE Staining

After fixing in Davidson solution for 24 h, the hepatopancreases were transferred into 70% ethanol. Paraffin-embedded hepatopancreas was sliced into sections of 5 μm thickness and used for HE staining. The histological changes were observed under a light microscope (Olympus BX60, Olympus, Tokyo, Japan).

### 2.9. Statistical Analysis

The data are expressed in the form of the mean ± SD. SPSS 19.0 (IBM Corp., Armonk, NY, USA) was used for the statistical analysis of the data. One-way analysis of variance (ANOVA) and Duncan’s multiple range test were used to analyze the significant differences among different groups. The main effects of florfenicol and quercetin and their combinations in terms of immune parameters, Vibrio density, protection ratio, and cumulative mortality were assessed using two-way ANOVA.

## 3. Results

### 3.1. Survival and RPS of Shrimp

**Figure 1:**
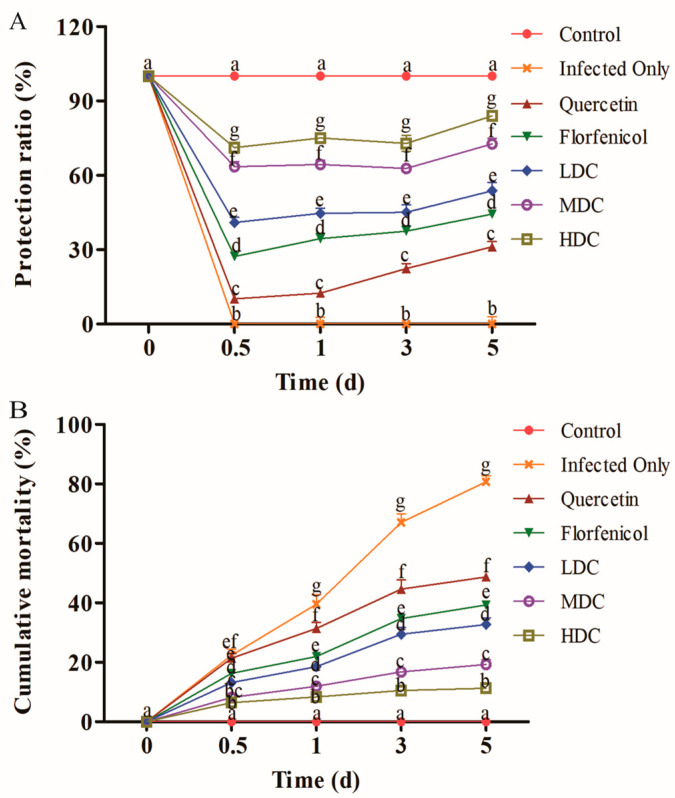
The (**A**) protection rates and (**B**) cumulative mortality of VP_AHPND_-infected *L. vannamei* in each experimental group at different times after drug treatment. One-way ANOVA and Duncan’s multiple range test were used to analyze the significant differences among different groups, and different letters represent significant differences between groups (*p* < 0.05).

During the experiments, the control shrimp showed no disease symptoms. The florfenicol and quercetin only groups showed higher survival than the VP_AHPND_ infection only group (*p* < 0.05; Figure 1). The shrimp survival rates and RPS of the drug combination groups were higher than those after treatment with either drug alone (*p* < 0.05; Figure 1). Moreover, the survival rate and RPS increased drug dose-dependently.

### 3.2. Hepatopancreatic VP_AHPND_ Clearance

**Figure 2:**
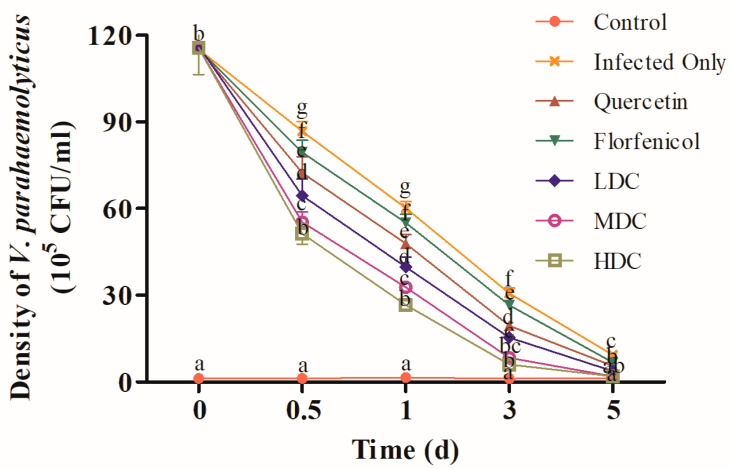
The VP_AHPND_ density in the hepatopancreas of *L. vannamei* at different times after drug treatment. One-way ANOVA and Duncan’s multiple range test were used to analyze the significant differences among different groups, and different letters represent significant differences between groups (*p* < 0.05).

The VP_AHPND_ density in the hepatopancreas of the infection only, florfenicol, quercetin, LDC, MDC, and HDC groups decreased by 24.65%, 31.07%, 37.35%, 44.59%, 52.38%, and 55.74% respectively at 0.5 dpi, and decreased by 47.75%, 52.27%, 58.59%, 65.80%, 71.83%, and 76.94%, respectively, at 1 dpi. At 3 dpi, the hepatopancreatic VP_AHPND_ density of the infection only, florfenicol, quercetin, LDC, MDC, and HDC groups decreased to 30.63, 26.47, 19.42, 15.32, 8.46, and 5.97 × 10^5^ CFU/mL, respectively. At 5 dpi, VP_AHPND_ was almost completely cleared from the shrimp hepatopancreas in the MDC and HDC groups (Figure 2).

### 3.3. THC and HEM Levels and Antimicrobial Activity in Shrimp

**Figure 3:**
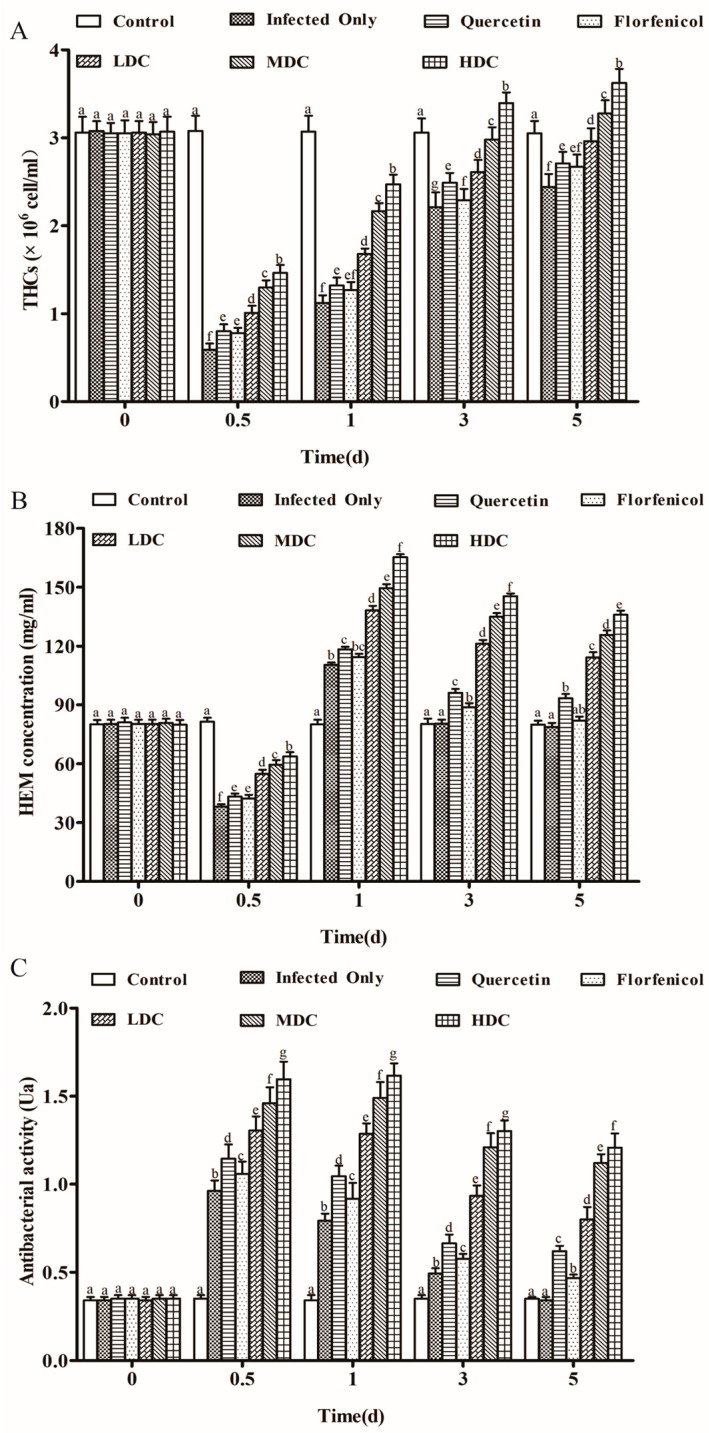
The THC levels (**A**), HEM levels (**B**), and antibacterial activity (**C**) in *L. vannamei* at different times following drug treatment. One-way ANOVA and Duncan’s multiple range test were used to analyze the significant differences among different groups, and different letters represent significant differences between groups (*p* < 0.05).

At 0.5 dpi, the THC level reached its lowest value in the VP_AHPND_ infection only group. During the experiment, the THC levels in the infection only group, florfenicol only group, and quercetin only group were always significantly lower than those in the control group. In the drug combination groups, the THC level showed a gradually increasing trend, approaching the level of the control group at the end of the experiment. Moreover, significantly lower THC levels were observed in the infection only group, the florfenicol only group, and the quercetin only group than those in the drug combination groups (Figure 3A). The shrimp HEM concentration showed a trend of first increasing and then decreasing over the course of the experiment, being the highest at 1 dpi. In the drug combination groups, the HEM concentration was significantly higher than that in the infection only group, the florfenicol group, and the quercetin group. Moreover, in the drug combination groups, the HEM levels increased dose-dependently (Figure 3B). In all experimental groups, the antibacterial activity in shrimp was highest at 0.5 and 1 dpi, and then decreased gradually. However, significantly higher antibacterial activities were observed in the drug combination groups than those in the infection only group, the florfenicol only group, and the quercetin only group throughout the experimental period (Figure 3C).

### 3.4. Immunity-Related Enzyme Activities in Acellular Hemolymph

**Figure 4:**
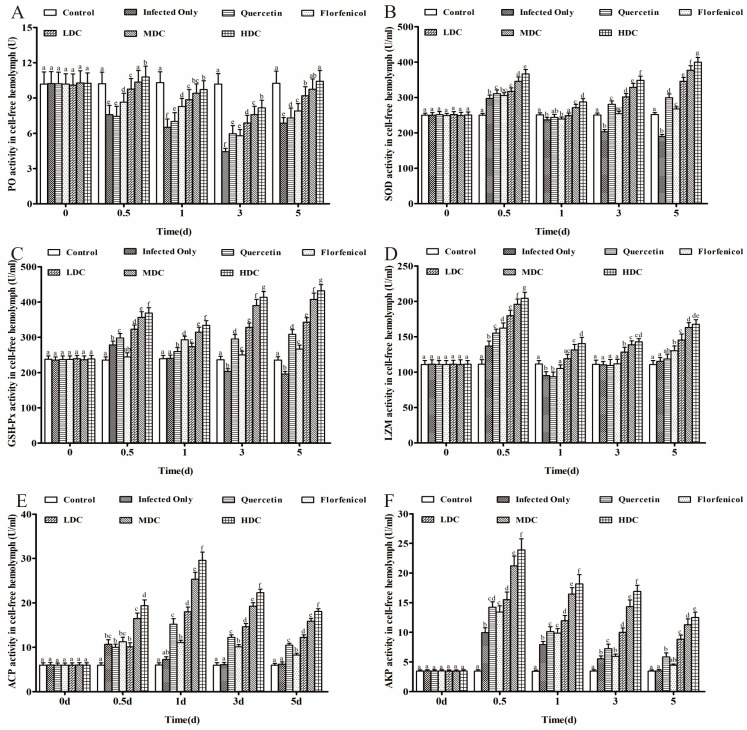
Immunity-related enzyme activities in drug-treated *L. vannamei*. (**A**) PO, (**B**) SOD, (**C**) GSH-Px, (**D**) LZM, (**E**) ACP, and (**F**) AKP in *L. vannamei* acellular hemolymph after different times of drug treatment. One-way ANOVA and Duncan’s multiple range test were used to analyze the significant differences among different groups, and different letters represent significant differences between groups (*p* < 0.05).

Compared with that in the control group, the PO activity in all the groups decreased after VP_AHPND_ infection. The drug combination groups showed higher PO activities than those in the infection only group, the florfenicol only group, and the quercetin only group. Moreover, in the drug combination groups, the PO activity increased dose-dependently (Figure 4A). After feeding with the drug-containing diets, significantly higher activities of SOD, GSH-Px, and LZM were observed in the drug combination groups compared with those in the infection only group, the florfenicol only group, and the quercetin only group (*p* < 0.05), and their activities also increased with increased drug doses (Figure 4B–D). In all the drug treatment groups, the activities of AKP and ACP peaked at 0.5 and 1 dpi, respectively. In the infection only group and the control group, the activities of ACP and AKP were similar at 3 and 5 dpi, respectively (*p* > 0.05; Figure 4E,F). However, compared with those in the control and infection only groups, higher ACP and AKP activities were observed in the drug treatment groups (*p* < 0.05). Furthermore, in the drug combination groups, the activities of ACP and AKP were higher compared with those after treatment with either drug alone, and the increase was drug dose-dependent.

### 3.5. Immunity-Related Gene Expression Profiles in Hemocytes

**Figure 5:**
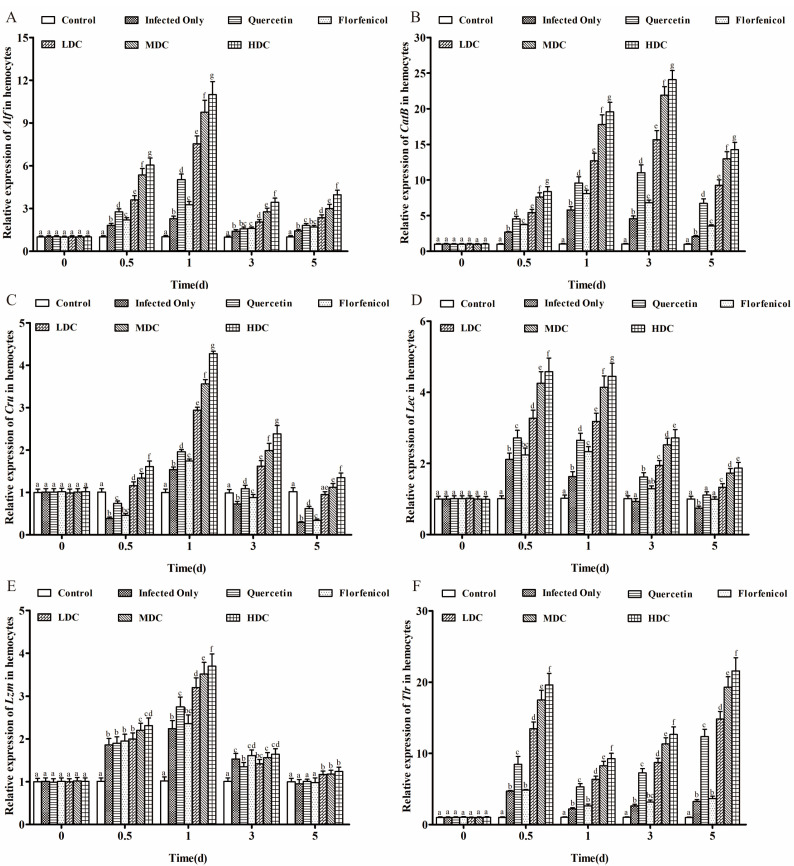
Immunity-related factors’ gene expression in drug-treated *L. vannamei.* (**A**) *Alf*, (**B**) *CatB*, (**C**) *Cru*, (**D**) *Lec*, (**E**) *Lzm*, and (**F**) *Tlr* in *L. vannamei* hemocytes at different times after drug treatment. One-way ANOVA and Duncan’s multiple range test were used to analyze the significant differences among different groups, and different letters represent significant differences between groups (*p* < 0.05).

At all experimental time points, *Alf* and *Cru* expression levels in hemocytes increased, peaking at 1 dpi. *Alf* and *Cru* expression levels were significantly higher in the drug combination groups compared with those after treatment with either drug alone throughout the experimental period (*p* < 0.05; Figure 5A,C). Except in the control group, the expression level of *CatB* in hemocytes increased in all the experimental groups. Moreover, in the drug combination groups, *CatB* expression was significantly higher than in the florfenicol and quercetin only groups (*p* < 0.05; Figure 5B). *Lec* expression in the drug treatment groups was significantly higher than that in the control group at all experimental time points, except that *Lec* expression in the florfenicol only group did not differ significantly from that in the control group at 5 dpi (*p* < 0.05; Figure 5D). From 0.5 to 3 dpi, the expression level of *Lzm* in all experimental groups increased significantly. Except in the drug combination groups, the expression of *Lzm* in the remaining experimental groups decreased to the level of the control group by 5 dpi (*p* < 0.05; Figure 5E). *Tlr* expression showed a trend of first decreasing and then increasing, reaching a peak at 5 dpi (Figure 5F). In addition, at all experimental time points, the expression levels of *Lec*, *Lzm*, and *Tlr* were significantly higher in the drug combination groups than in the florfenicol only and quercetin only groups.

### 3.6. Analysis of the Hepatopancreas Histology

**Figure 6:**
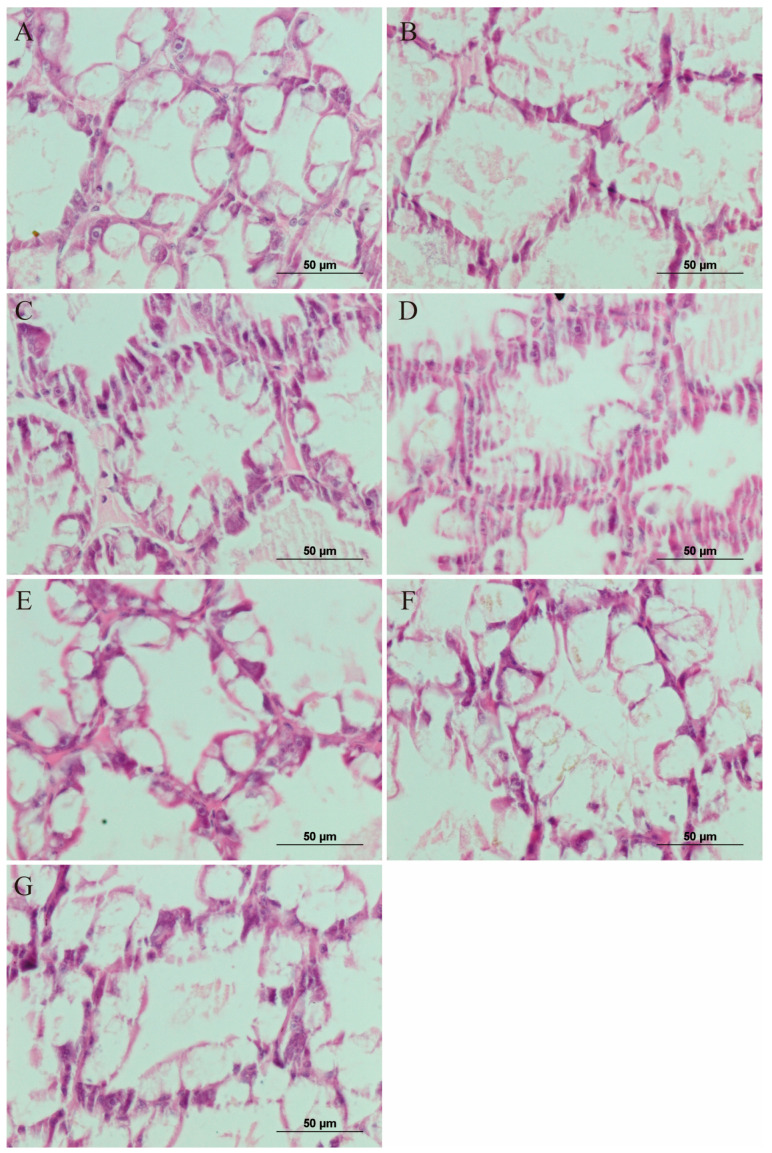
Changes in the histology of drug-treated *L. vannamei* hepatopancreases at 5 days. (**A**) Control, (**B**) infection only, (**C**) florfenicol, (**D**) quercetin, (**E**) LDC, (**F**) MDC, and (**G**) HDC group. Scale bar = 50 μm.

VP_AHPND_ infection disrupted the structural integrity of the hepatopancreatic tubules in comparison with that in the control group, accompanied by massive shedding and rupture of hepatopancreatic tubules epithelial cells. The use of either drug alone improved the hepatopancreatic tubules’ structural integrity, and increased the number of the epithelial cells in intact hepatopancreatic tubules. The combined drug treatment improved the structure of the hepatopancreas tubules to a greater extent than either drug alone, and showed combined drug dose-dependency (Figure 6).

Moreover, for the immune parameters, Vibrio density, RPS, and cumulative mortality, we observed significant interactions (*p* < 0.05) between florfenicol and quercetin in shrimp (Table 3).

## 4. Discussion

Virulent strains of VP_AHPND_ contain a 70 kbp plasmid (pVA1) that encodes a homolog of photorhabdus insect-associated (Pir) toxins PirA and PirB [3,5]. The Pir toxin secreted by VP_AHPND_ can damage shrimp hepatopancreatic cells [29]. VP_AHPND_ infection causes hepatopancreatic epithelial cells to be exfoliated or become necrotic, leading to shrimp hepatopancreatic atrophy, which is mostly fatal to shrimp [30].

For a long time, antibiotics have been the most commonly used method to treat bacterial infections in shrimp aquaculture. Unfortunately, the long-term, high-level use of antibiotics has led to drug residues and drug resistance. However, the combined use of traditional Chinese medicine with antibiotics, could not only reduce the problems caused by antibiotics, but also can enhance the immunity of the animals.

In the human diet, the main source of antioxidants is plant flavonoids which have various biological functions, such as anticancer, anti-inflammatory, and antibacterial abilities [31]. As a lipophilic compound, quercetin exists mainly in various fruit and vegetables in a glycosylated form, and is the most common flavonoid in human food [32]. In addition to its anticancer, antiviral, antibacterial, and anti-inflammatory effects, quercetin also has good antioxidant activity and free radical scavenging effects [33]. Previous studies have shown that quercetin is closely related to immune responses and disease resistance in aquatic animals [20,21,22]. Therefore, in this study, the anti-infective effect of quercetin combined with florfenicol in VP_AHPND_-infected *L. vannamei* was evaluated, and the combined effect of the two drugs was compared with the effect of quercetin or florfenicol used alone.

Accordingly, feeding shrimp with a diet containing quercetin and florfenicol effectively reduced the cumulative mortality of *L. vannamei* infected with VP_AHPND_. Moreover, quercetin and florfenicol in combination was more effective than either drug used alone. Flavonoids can enhance the immunity and resistance to infection of animals [22]. Therefore, the above results might reflect the antibacterial function of quercetin and its non-specific immunity enhancement in *L. vannamei*.

As invertebrates, shrimp rely mainly on humoral and cellular innate immunity to recognize and kill invading pathogens [34,35]. In this study, we observed that the clearance rate of VP_AHPND_ was higher in the drug combination groups and the florfenicol and quercetin alone groups than that in the infection only group. Moreover, the drug combination groups had the best effect among all the treatment groups, indicating that a diet comprising quercetin combined with florfenicol resulted in improved shrimp resistance to VP_AHPND_ infection, which might benefit from the antibacterial activity of florfenicol and the immunological activity of quercetin.

In shrimp that have been exposed to infectious pathogens, the risk of secondary infection might be increased by decreased THC levels [36]. Our results revealed a quercetin and florfenicol combination-mediated increase in shrimp THC levels. The increase in the THC level indicated enhanced disease resistance of shrimp, suggesting that quercetin might enhance the immunity of shrimp by promoting the proliferation and phagocytosis of hemocytes in shrimp. Similar results were observed for THC levels when Sargasso polysaccharide was fed to *Fenneropenaeus chinensis* and polysaccharides derived from *Durio zibethinus* were fed to giant tiger shrimp [37,38].

HEM enhances the defense ability of shrimp against pathogens with its antibacterial and antiviral activities [39]. Previous research showed that the concentration of HEM decreased after shrimp were infected with pathogens [40]. In this study, the HEM level in the drug combination groups was markedly higher than those in the groups treated with either drug alone; therefore, the drug combination could significantly enhance the ability of shrimp to resist pathogenic infection.

The antimicrobial peptides contained in the hemolymph of shrimp have antibacterial activity. An increased antimicrobial peptide content can enhance the shrimp’s ability to resist pathogenic infection [41]. In this study we found that, compared with the infection only group, the antibacterial activities in the drug treatment groups were significantly higher. More importantly, the drug combination groups showed significantly higher antibacterial activity compared with those in the groups treated with either drug alone. This means that the drug combination induced higher antibacterial activity in shrimp.

The main immune-related enzymes in shrimp are PO, SOD, GSH-Px, LZM, ACP, and AKP. Previous studies showed that, after infection with *Vibrio alginolyticus*, the PO activity in shrimp first decreased and then gradually returned to normal levels, and the PO activity in VP_AHPND_-infected shrimp showed a similar trend in this study [42]. In addition, a previous study found that the levels of SOD and GSH-Px in shrimp were significantly increased after *L. vannamei* were infected with WSSV [43]. The activity of LZM in shrimp increased significantly after *Portunus trituberculatus* were infected with *Vibrio alginolyticus*, and the activities of ACP and AKP were elevated after *Exopalaemon carinicauda* were infected with WSSV [44]. The above studies showed that immune-related enzymes in shrimp play an important role in responding to pathogenic infections. Our study showed that the activities of the six enzymes were higher in the LDC group than in the groups treated with either drug alone, indicating that, under the premise of achieving better therapeutic effects, the use of the florfenicol could be reduced when quercetin and florfenicol were used in combination.

The activation of an innate immune response is marked by the pattern recognition receptors (PRRs) recognizing pathogens [45]. As an important PRR, lectin can enhance the phagocytic activity of hemocytes and the agglutination reaction of bacteria resulting from the invasion of VP_AHPND_ [46,47]. In this study, *Lec* expression increased in shrimp hemocytes post VP_AHPND_ infection. Moreover, the *Lec* level in the groups treated with the combined drugs was significantly higher than that in the groups treated with either drug alone, which indicated that the shrimp in the drug combination groups had stronger disease resistance. In invertebrate innate immunity, the Toll signaling pathway is the most important signaling pathway that participates in pathogen identification and defense. The transmembrane glycoprotein, TLR, is an important functional factor in the Toll signaling pathway. In innate immunity, the PRR TLR plays an important role in the identification of pathogens in innate immunity. As a transmembrane glycoprotein, TLR has a transmembrane domain and an extracellular N-terminal region that is rich in leucine repeat sequences (LRRs). LRRs recognize pathogen-related molecular patterns (PAMPs), while the intracellular C-terminus contains a Toll/interleukin-1 receptor (TIR) domain that is required for downstream signal transduction [48,49]. ALF, crustin, and LZM are important AMPs with broad-spectrum antimicrobial effects [50,51,52]. Thus, TLR, ALF, crustin, and LZM all play important roles in shrimp pathogen resistance. Interestingly, the expression levels of *Tlr*, *Alf*, *Cru*, and *Lzm* in the drug combination groups were higher than those in the groups treated with either drug alone. CatB is a cathepsin and is involved in antigen processing in antigen-presenting cells [53,54]. A previous study showed that the *CatB* expression in WSSV-infected *F. chinensis* increased [55]. In this study, hemocyte *CatB* expression was also upregulated after *L. vannamei* infection with VP_AHPND_. Moreover, *CatB* expression in the drug combination groups exceeded that in the groups treated with either drug alone, which meant that the drug combination groups have higher anti-infection ability.

The shrimp’s hepatopancreas is VP_AHPND_’s target organ. After infection with VP_AHPND_, the hepatopancreatic tubule epithelial cells are ruptured, and their nuclei become deformed, condensed, or disappear. The hepatopancreas will undergo diffuse necrosis, eventually leading to hepatopancreatic atrophy with VP_AHPND_ infection [4,56]. Herein, we demonstrated an improved hepatopancreatic structure in the shrimp treated with the drugs compared with those only infected with VP_AHPND_. Moreover, the hepatopancreatic structure showed more improvement in the drug combination groups than in the groups treated with either drug alone. This indicated that shrimp resistance to VP_AHPN_ was enhanced by treatment with the drugs in combination. This might be related to the antibacterial activity and immune enhancement function of quercetin.

## 5. Conclusions

In conclusion, compared with florfenicol or quercetin used alone, the combined use of the two drugs significantly improved the survival rate, drug protection, bacterial clearance, and immune parameters including the antibacterial actions, as well as the THC and HEM levels, the GSH-Px, PO, SOD, LZM, ACP, and AKP enzyme activities in the hemolymph, and the *Alf*, *CatB*, *Cru*, *Lec*, and *Lzm* mRNA expression in *L. vannamei* hemocytes within 5 dpi. The hepatopancreas histology was also improved in the drug combination groups. Moreover, florfenicol and quercetin interacted significantly, affecting the above indicators. The results showed that shrimp disease resistance improved when florfenicol and quercetin were used in combination. Therefore, under the premise of obtaining better therapeutic effects, a combination with quercetin can reduce the use of florfenicol and reduce antibiotic pollution in shrimp and the environment, thereby decreasing the occurrence of drug resistance.

## Figures and Tables

**Table 1:** The formula composition of the diets in the experiment.

	Groups						
	Control	Infected Only	Quercetin	Florfenicol	LDC	MDC	HDC
Ingredients							
Fish meal ^a^ (g/kg)	200.0	200.0	200.0	200.0	200.0	200.0	200.0
Wheat glutens ^a^ (g/kg)	300.0	300.0	300.0	300.0	300.0	300.0	300.0
Wheat meal ^a^ (g/kg)	200.0	200.0	200.0	200.0	200.0	200.0	200.0
Cellulose (g/kg)	180.0	180	179.6	179.99	179.79	179.59	179.17
Fish oil (g/kg)	25.0	25.0	25.0	25.0	25.0	25.0	25.0
Soybean oil (g/kg)	25.0	25.0	25.0	25.0	25.0	25.0	25.0
Soybean phospholipids (g/kg)	20.0	20.0	20.0	20.0	20.0	20.0	20.0
Gelatin (g/kg)	20.0	20.0	20.0	20.0	20.0	20.0	20.0
Choline chloride (g/kg)	10.0	10.0	10.0	10.0	10.0	10.0	10.0
Vitamin mix ^b^ (g/kg)	10.0	10.0	10.0	10.0	10.0	10.0	10.0
Mineral mix ^c^ (g/kg)	10.0	10.0	10.0	10.0	10.0	10.0	10.0
Quercetin (mg/kg)	0	0	400	0	200	400	800
Florfenicol (mg/kg)	0	0	0	15	7.0	15	30
Proximate nutrient composition (as fed)							
Crude protein (g/kg)	431.0	431.0	431.0	431.0	431.0	431.0	431.0
Crude fat (g/kg)	73.0	73.0	73.0	73.0	73.0	73.0	73.0
Crude ash (g/kg)	68.0	68.0	68.0	68.0	68.0	68.0	68.0
Total energy (kJ/g)	16.44	16.44	16.44	16.44	16.44	16.44	16.44

^a^ Fish meal: Crude protein, 689.9 g/kg dry matter, crude fat, 78.1 g/kg dry matter; wheat glutens: crude protein, 790.5 g/kg dry matter; crude fat, 1.8 g/kg dry matter; wheat meal: crude protein, 165.0 g/kg dry matter; crude fat, 15.8 g/kg dry matter. ^b^ Vitamin mixture (mg/kg diet): Riboflavin, 45.0 mg; thiamine, 25.0 mg; vitamin K3, 10.0 mg; inositol, 800.0 mg; pyridoxine hydrochloride, 20.0 mg; vitamin B12, 0.1 mg; calcium pantothenate, 60.0 mg; biotin, 1.2 mg; vitamin A, 32.0 mg; vitamin D, 5.0 mg; nicotinic acid, 200.0 mg; folic acid, 20.0 mg; vitamin E, 120.0 mg. ^c^ Mineral mix (mg/kg diet): KI, 0.8 mg; NaF, 2.0 mg; NaCl, 100.0 mg; MgSO_4_, 200.0 mg; CuSO_4_·5H_2_O, 10.0 mg; CoCl_2_·6H_2_O, 50.0 mg; ZnSO_4_, 50.0 mg; Fe_2_(SO_4_)_3_, 80.0 mg; Ca(H_2_PO_4_)_2_, 3000.0 mg.

**Table 2:** Primer information for qPCR used in the experiment.

Primer	Primer Sequence (5′-3′)	GenBank Accession Number
*Tlr*-F	TGAGAGATGCCCACTGCCTG	DQ923424.1
*Tlr*-R	CGCTTGAAGGTTTGTGAGGGAG	
*Alf*-F	TGTTCCTGGTGGCACTCTTC	GQ227486.1
*Alf*-R	GTCTCCTCGTTCCTCCACAG	
*Cru*-F	AACCAGAGACACCTGTTGGC	AY488497.1
*Cru*-R	AGAATGAGGGAGGCTTGCAC	
*Lzm*-F	TCGAGTCGTCCTTCAACACG	AF425673.1
*Lzm*-R	AGACGTTCTTGCCGTAGTCG	
*Lec*-F	CGGGATCCATGAAGTTCCTAGCGCCG	EF583939.1
*Lec*-R	CGCTCGAGTATATTTCTTGAGGCAAAT	
*CatB*-F	CCTCTGTGGTTTTGGATGTA	GU571199.1
*CatB*-R	GATGCTGTATGCTTTGCCTC	
*β-actin-*F	AGTAGCCGCCCTGGTTGT	AF300705.2
*β-actin*-R	AGGATACCTCGCTTGCTCT	

**Table 3:** Two-way ANOVA analysis of cumulative mortality, protection ratio, Vibrio density and immune parameters with florfenicol and quercetin.

		0 d		0.5 d		1 d		3 d		5 d	
		F	*p*	F	*p*	F	*p*	F	*p*	F	*p*
	f	0.005	0.196	93.267	0.000	84.533	0.003	270.506	0.002	597.818	0.000
Cumulative mortality	q	0.030	0.188	37.363	0.000	51.956	0.001	166.258	0.001	367.430	0.000
	f × q	0.050	0.185	6.039	0.003	0.894	0.009	2.860	0.014	6.320	0.027
	f	0.680	0.144	2.556	0.026	87.402	0.013	279.687	0.001	618.109	0.000
Protection ratio	q	0.115	0.176	29.076	0.000	54.162	0.001	173.319	0.001	383.035	0.000
	f × q	0.060	0.183	1.005	0.033	7.064	0.027	22.605	0.001	4.485	0.043
	f	0.009	0.847	254.365	0.000	126.111	0.000	336.524	0.000	9.680	0.010
Vibrio density	q	0.001	0.888	101.898	0.000	43.015	0.000	88.474	0.000	4.044	0.062
	f × q	0.000	0.908	16.471	0.003	10.322	0.009	19.093	0.002	0.225	0.575
	f	0.077	0.725	6.970	0.022	1.955	0.165	4.817	0.455	4.815	0.045
THCs	q	0.077	0.725	79.297	0.000	0.575	0.408	13.967	0.004	7.194	0.021
	f × q	0.187	0.816	2.741	0.110	7.625	0.018	1.552	0.207	0.951	0.305
	f	0.004	0.865	60.531	0.000	159.490	0.000	148.126	0.000	55.695	0.000
HEM	q	0.298	0.561	185.007	0.000	454.173	0.000	731.756	0.000	393.622	0.000
	f × q	0.024	0.805	18.015	0.002	27.887	0.001	8.339	0.015	26.103	0.001
	f	0.207	0.615	11.409	0.007	0.007	0.846	45.589	0.000	37.403	0.000
Antibacterial activity	q	0.207	0.615	9.973	0.010	1.385	0.229	199.579	0.000	702.337	0.000
	f × q	0.207	0.615	6.273	0.027	0.561	0.414	15.581	0.003	25.974	0.001
	f	0.001	0.890	0.360	0.497	1.125	0.271	13.069	0.005	7.017	0.022
PO activity	q	0.007	0.853	18.273	0.002	22.375	0.001	82.919	0.000	42.334	0.000
	f × q	0.011	0.841	1.504	0.213	0.115	0.665	4.471	0.039	0.204	0.589
	f	0.150	0.656	0.225	0.575	7.653	0.018	82.949	0.000	27.931	0.001
SOD activity	q	0.025	0.802	3.792	0.068	10.656	0.008	182.416	0.000	38.814	0.000
	f × q	0.013	0.834	1.172	0.263	10.821	0.008	0.126	0.654	3.592	0.075
	f	0.021	0.812	16.397	0.003	17.254	0.002	94.145	0.000	114.238	0.000
GSH-Px activity	q	0.008	0.852	25.453	0.001	34.565	0.000	187.856	0.000	221.828	0.000
	f × q	0.050	0.762	10.680	0.008	14.396	0.004	5.835	0.032	2.126	0.150
	f	0.006	0.858	9.263	0.012	18.371	0.002	13.245	0.005	26.161	0.001
LZM activity	q	0.000	0.898	20.739	0.001	36.595	0.000	31.422	0.000	102.075	0.000
	f × q	0.007	0.853	1.969	0.163	7.939	0.016	11.225	0.007	11.276	0.007
	f	0.000	0.897	12.535	0.006	112.852	0.000	161.979	0.000	110.784	0.000
ACP activity	q	0.000	0.897	35.606	0.000	349.793	0.000	710.563	0.000	499.029	0.000
	f × q	0.008	0.850	11.229	0.007	27.126	0.001	55.475	0.000	53.592	0.000
	f	0.002	0.879	23.603	0.001	44.391	0.000	75.945	0.000	71.283	0.000
AKP activity	q	0.019	0.818	68.846	0.000	112.016	0.000	150.743	0.000	170.969	0.000
	f × q	0.002	0.879	16.289	0.003	18.241	0.002	52.947	0.000	39.405	0.000
	f	0.116	0.686	54.434	0.000	310.115	0.000	55.365	0.000	55.063	0.000
*Alf* expression	q	0.116	0.686	101.094	0.000	1168.187	0.000	168.119	0.000	124.353	0.000
	f × q	0.013	0.834	37.731	0.000	238.163	0.000	41.080	0.000	39.807	0.000
	f	0.052	0.758	95.303	0.000	94.525	0.000	138.370	0.000	95.035	0.000
*CatB* expression	q	0.052	0.758	528.251	0.000	214.873	0.000	513.988	0.000	389.612	0.000
	f × q	0.000	0.909	92.325	0.000	84.915	0.000	127.617	0.000	89.241	0.000
	f	0.052	0.758	69.401	0.000	108.255	0.000	68.537	0.000	69.328	0.000
*Cru* expression	q	0.052	0.758	200.568	0.000	179.874	0.000	199.446	0.000	135.883	0.000
	f × q	0.000	0.909	39.513	0.000	52.957	0.000	40.079	0.000	46.409	0.000
	f	0.000	0.909	55.345	0.000	66.504	0.000	70.715	0.000	82.324	0.000
*Lec* expression	q	0.000	0.909	106.820	0.000	245.946	0.000	297.324	0.000	227.333	0.000
	f × q	0.052	0.758	40.208	0.000	52.042	0.000	57.150	0.000	66.246	0.000
	f	0.013	0.833	8.292	0.015	8.615	0.014	5.177	0.040	1.989	0.161
*Lzm* expression	q	0.116	0.685	7.275	0.020	65.155	0.000	12.109	0.005	0.405	0.476
	f × q	0.116	0.685	3.732	0.070	4.846	0.043	1.791	0.180	1.108	0.275
	f	0.000	0.909	73.174	0.000	140.643	0.000	71.193	0.000	49.602	0.000
*Tlr* expression	q	0.045	0.767	1291.659	0.000	1349.896	0.000	516.204	0.000	474.346	0.000
	f × q	0.045	0.767	67.100	0.000	93.076	0.000	40.728	0.000	36.800	0.000

Note: f, florfenicol; q, quercetin; f × q indicates interactive effect of florfenicol and quercetin. F and *p* refer to F and *p* values of two-way ANOVA analysis respectively.

## Data Availability

Not applicable.
